# Twenty Years of Medically-Attended Pediatric Varicella and Herpes Zoster in Ontario, Canada: A Population-Based Study

**DOI:** 10.1371/journal.pone.0129483

**Published:** 2015-07-15

**Authors:** Anne E. Wormsbecker, Jun Wang, Laura C. Rosella, Jeffrey C. Kwong, Chi Yon Seo, Natasha S. Crowcroft, Shelley L. Deeks

**Affiliations:** 1 Public Health Ontario, Toronto, Ontario, Canada; 2 Division of Paediatric Medicine, Hospital for Sick Children, Toronto, Ontario, Canada; 3 Dalla Lana School of Public Health, University of Toronto, Toronto, Ontario, Canada; 4 Institute for Clinical Evaluative Sciences, Toronto, Ontario, Canada; Public Health England, UNITED KINGDOM

## Abstract

**Objective:**

To determine if reductions in medically-attended pediatric varicella and herpes zoster occurred in Ontario, Canada, after publicly-funded varicella immunization was implemented in 2004.

**Methods:**

For fiscal years (FY) 1992-2011, we examined data on varicella and herpes zoster physician office visits, emergency department (ED) visits, hospitalizations (including for varicella-associated skin and soft tissue infections [SSTI]), and intensive care unit (ICU) admissions, among those aged <18 years. The pre-vaccine, privately-available, and vaccine program eras were FY1992-1998, FY1999-2003, and FY2004-2011, respectively. We used Poisson regressionand Kruskal-Wallis tests (all at the p<0.05 level of significance), and compared rates using incidence rate ratios (IRRs) and 95% confidence intervals (CIs).

**Results:**

Incidence of varicella office visits declined over the study period from a high of 25.1/1,000 in FY1994 to a low of 3.2/1,000 in FY2011. ED visits and hospitalizations followed similar patterns of decreasing rates later in the study period. IRRs comparing the vaccine program versus pre-vaccine eras were 0.29 (95%CI: 0.26-0.32) for office visits, 0.29 (95%CI: 0.21-0.40) for ED visits, and 0.41 (95%CI: 0.10-1.69) for hospitalizations. Annual declines in varicella office visits were 7.7%, 9.1%, 8.4%, and 8.4% per year among children aged <1 year, 1-4 years, 5-11 years, and ≥12 years, respectively (all p<0.001). Age-specific rates of varicella-associated SSTI declined significantly among children <12 years (p<0.001) and rates of ICU admissions decreased significantly for children <1 year (p = 0.02). (p<0.001) over the study period. For children aged 5-17 years, herpes zoster office visits decreased whereas ED visits increased (both p<0.001) and there was a small, non-significant (p = 0.07), decrease in hospitalizations.

**Conclusion:**

Medically-attended varicella decreased during the study period, particularly since varicella vaccine was publicly-funded. Results suggest immunization program-related changes in varicella epidemiology, including herd effects, demonstrated by reductions in varicella in program-ineligible age groups. We did not observe a consistent impact on herpes zoster.

## Introduction

Varicella is a common childhood illness with the potential for serious complications, even among healthy individuals [1]. Internationally, varicella vaccines have been available since the mid-to-late-1990s and studies conducted in settings of routine childhood varicella immunization have demonstrated reductions in the incidence of varicella and related outcomes [1–5]. Although varicella immunization programs have been effective, varicella-related surveillance and research have identified challenges such as limited impact of varicella immunization through private purchase rather than publicly-funded programs, including in our own province of Ontario [6,7]; the inability of single-dose programs to prevent outbreaks [8,9,10]; and conflicting data regarding the impact of childhood varicella vaccine on pediatric herpes zoster [1, 11–14].

Although varicella vaccine was approved for use in Canada in December 1998 (Varivax, Merck Canada, followed by Varilrix, GlaxoSmithKline, in October 1999), Ontario did not offer publicly-funded immunization until September 2004, later than some other Canadian provinces and much later than its widespread use in the United States after licensure in 1995 [3,5]. Since then, children born in 2000 and later have been eligible to receive publicly-funded catch-up or routine childhood varicella vaccine. The routine program began with a single dose at 15 months of age., In 2010, Canada’s National Advisory Committee on Immunization (NACI) recommended two doses of varicella, based on data suggesting two doses would reduce the number of breakthrough cases of varicella and prevent outbreaks, and a varicella booster for Ontario children aged 4–6 years was added in August 2011 [15].

Ontario’s 13.7 million (2013) inhabitants comprise approximately 40% of Canada’s population [16] and medical services are funded by the universal, single-payer Ontario Health Insurance Plan (OHIP). In general, publicly-funded vaccines are purchased from manufacturers by the provincial government and distributed through local public health units to health care providers who immunize their patients. Records are kept by providers and on cards given to vaccinated individuals but the province does not have an immunization registry. Public Health Ontario, an arm’s length government agency, carries out vaccine preventable disease surveillance. Given the need to monitor changes in varicella and herpes zoster epidemiology in the setting of our routine varicella immunization program, this study had four objectives. We sought, first, to describe health care utilization attributed to varicella in Ontario with population-based data and second, to use this data to identify changes to medically-attended varicella over time. Third, with 7.5 years of observation since public funding, we looked for further immunization program-related changes in varicella epidemiology, particularly changes in disease severity and complications, age distribution, and indirect/herd effects. Although we were not able to assess the impact of Ontario’s recent change to a two-dose varicella vaccine program, our final objective was to study temporal trends in herpes zoster among Ontario children.

## Materials and Methods

### Ethics Statement

This study involved the analysis of coded health care administrative data and was approved by the University of Toronto and Sunnybrook Health Sciences Centre Research Ethics Boards.

### Methods

Coded administrative data from OHIP (physician billing claims) and the Canadian Institute for Health Information (CIHI) (hospitalization discharge abstracts) are among the databases available from the Institute for Clinical Evaluative Sciences (ICES), Toronto, Ontario. Public deposition of ICES data is not legally permitted.

We used these health administrative data, organized by April to March fiscal year (FY), to assess trends in physician office visits, emergency department (ED) visits, and hospitalizations for varicella among children and youth aged <18 years from April 1992 to March 2012 (FYs 1992–2011). ED data were only available until the end of March 2011 (FY 2010). For office and ED visits, we used OHIP physician billing claims with the diagnostic code for varicella (052). We extracted hospitalizations with a Most Responsible Diagnosis (“the one diagnosis or condition that is accountable for the greatest portion of the length of stay or greatest use of resources” [17]) of varicella (*International Classification of Diseases*, *Ninth Revision* [ICD-9] codes 052.0, 052.1, 052.2, 0.52.7, 052.8, 052.9, or ICD, *Tenth Revision* [ICD-10] codes B01.0, B01.1, B01.2, B01.8, B01.9) from the CIHI Discharge Abstract Database (DAD).

We studied intensive care unit (ICU) admissions as one marker of the occurrence of severe illness or varicella complications. Because skin and soft tissue infection (SSTI) is the most common complication of childhood varicella [4], hospitalization for varicella-associated SSTI was the other complication evaluated. We used both the CIHI DAD (varicella codes as above) and OHIP databases to identify varicella-related ICU admissions, using the previously validated methods developed by Scales and colleagues [18,19]. OHIP ICU codes were G557, G558, G559, G400, G401, G402, G405, G406, G407, and C101. Because the ICD-9 diagnostic codes in the DAD were limited to a maximum of four digits whereas the ICD-9 codes for necrotizing fasciitis and Group A streptococcal infection contain five digits, we were only able to examine SSTIs using ICD-10-coded data(four digits) from FY2002-2011. SSTIs were defined as the simultaneous presence of a varicella code (not limited to most responsible diagnosis) *and* one of cellulitis, necrotizing fasciitis, or group A streptococcal (GAS) infection (excluding GAS pharyngitis) (S1 Table).

We obtained data on medically-attended herpes zoster using the same methods as for varicella but with herpes zoster as the diagnostic code (OHIP: 053; ICD-9: B053.0–053.9; ICD-10: B02.0-B02.9). Since herpes zoster is uncommon in young children [20] and data cleaning revealed a number of biologically implausible episodes of medically-attended herpes zoster among young children e.g. 41–112 physician office visits per year for children < 1 year old when near zero would be expected), results presented are primarily limited to herpes zoster among children aged 5–17 years to most conservatively optimize specificity by removing any varicella cases who may have been misclassified as herpes zoster. We excluded healthcare encounters where varicella and herpes zoster diagnostic codes coexisted.

We used pediatric (aged <18 years) demographic data from Statistics Canada obtained through intelliHealth Ontario to calculate crude incidence by health care setting (office, ED, hospital ward, ICU). We only considered the first such incidence for each outcome over the study period. In situations where fewer than six cases occurred per year, incidences were calculated accurately but case counts are expressed as < 6, for privacy reasons. The pre-vaccine era was FY1992 to FY1998, the privately-available vaccine era was FY1999 to FY2003, and the vaccine program era was FY2004 to FY2011. There were 5 months in the vaccine program era when the vaccine was not publicly funded, but this date was chosen as a conservative measure.

We statistically assessed yearly changes in incidence using a Poisson regression model with rate as the outcome and population as the denominator. We calculated incidence rate ratios (IRRs) and 95% confidence intervals (CIs) across the three eras, using a categorical time variable in the model. The Kruskal-Wallis test evaluated statistical significance of differences in median age. All statistical analyses were carried out with a threshold of p<0.05 for significance and were conducted using SAS, Version 9.3 (The SAS Institute, Cary, NC).

Although the primary purpose of our analysis was to examine the program impact on rates of health care utilization due to varicella, we conducted secondary analyses using age-adjusted rates to evaluate the impact that any changing age-structure over the time period had on our findings. Secondary analyses included calculation of age-standardized annual rates of all outcomes (standardized to the 1991 Canadian population) and assessment of changes in incidence of herpes zoster outcomes including children <5 years. The secondary herpes zoster analyses were conducted to determine if the age restriction (5–17 years) we chose to improve specificity yielded different results from study of all children <18 years.

## Results

### Medically-attended varicella

Between FY1992 and FY2011 (FY2010 for ED visits), we observed 600,208 incident physician office visits, 55,472 incident ED visits, and 2,701 incident hospitalizations for varicella among Ontario children. In each of these respective clinical settings, the proportion male was 51.2%, 51.7%, and 56.2%. The highest age-specific rates of medically-attended varicella were among children aged 1–4 years for office visits and ED visits (2122.4 and 210.4 per 100,000 population, respectively), whereas the highest age-specific rate of hospitalization was among infants aged <1 year (17 per 100,000 population). Although infants accounted for only 5.9% of office visits and 9.1% of ED visits, they made up 17.4% of hospitalizations.

### Immunization program-related changes

#### a) Trends in varicella epidemiology

**Fig 1:**
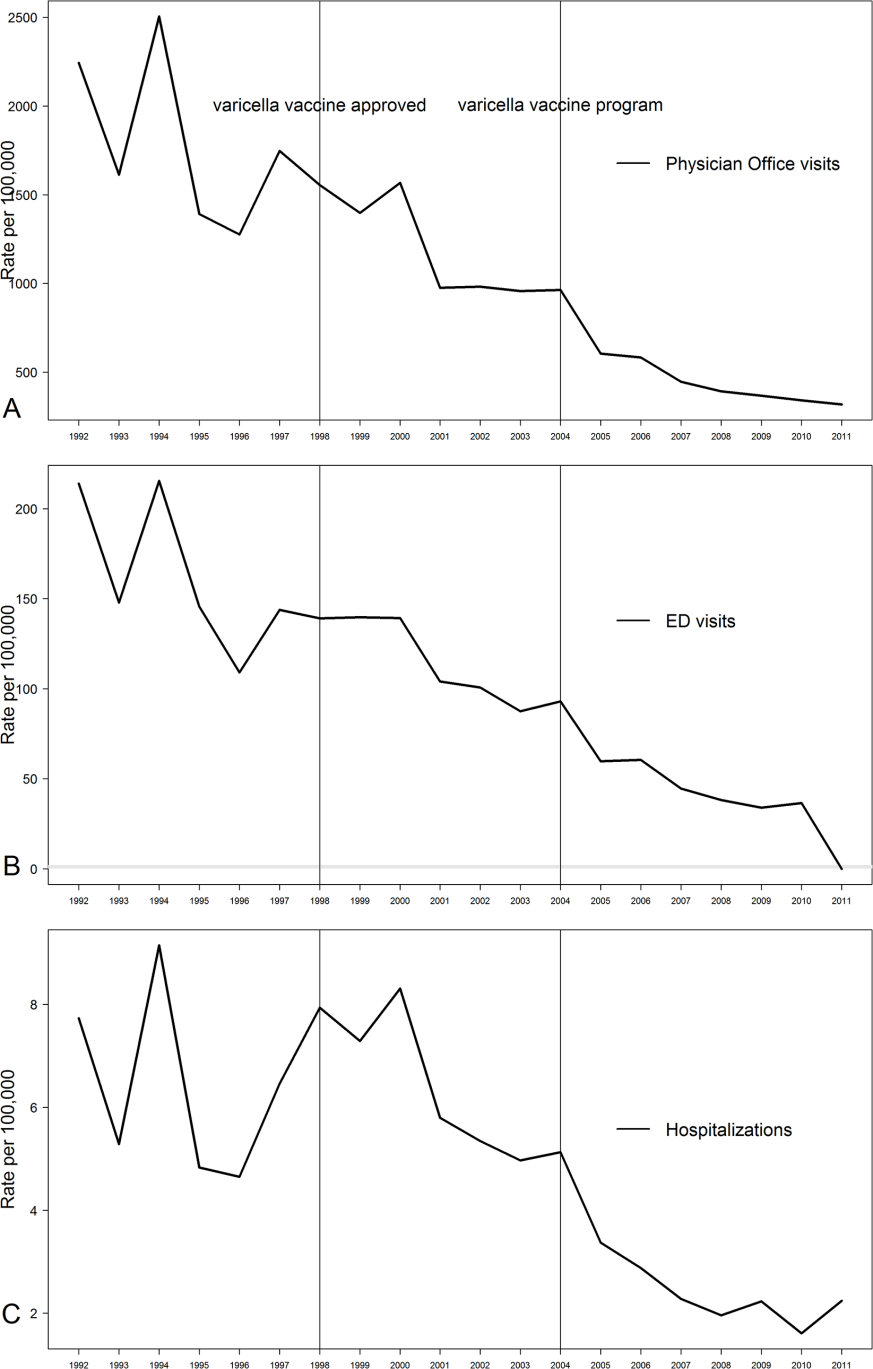
Incidence of varicella a) physician office visits (n = 600,208), b) ED visits (n = 55,472), and c) hospitalizations (n = 2,701) among Ontario children by fiscal years 1992–2011.

Crude annual incidence rates of pediatric varicella office visits, ED visits, and hospitalizations are shown in Fig 1A, 1B, and 1C. By Poisson regression, all decreased significantly over time (all p<0.001), and the analyses conducted with age-adjusted rates were no different.

**Fig 2:**
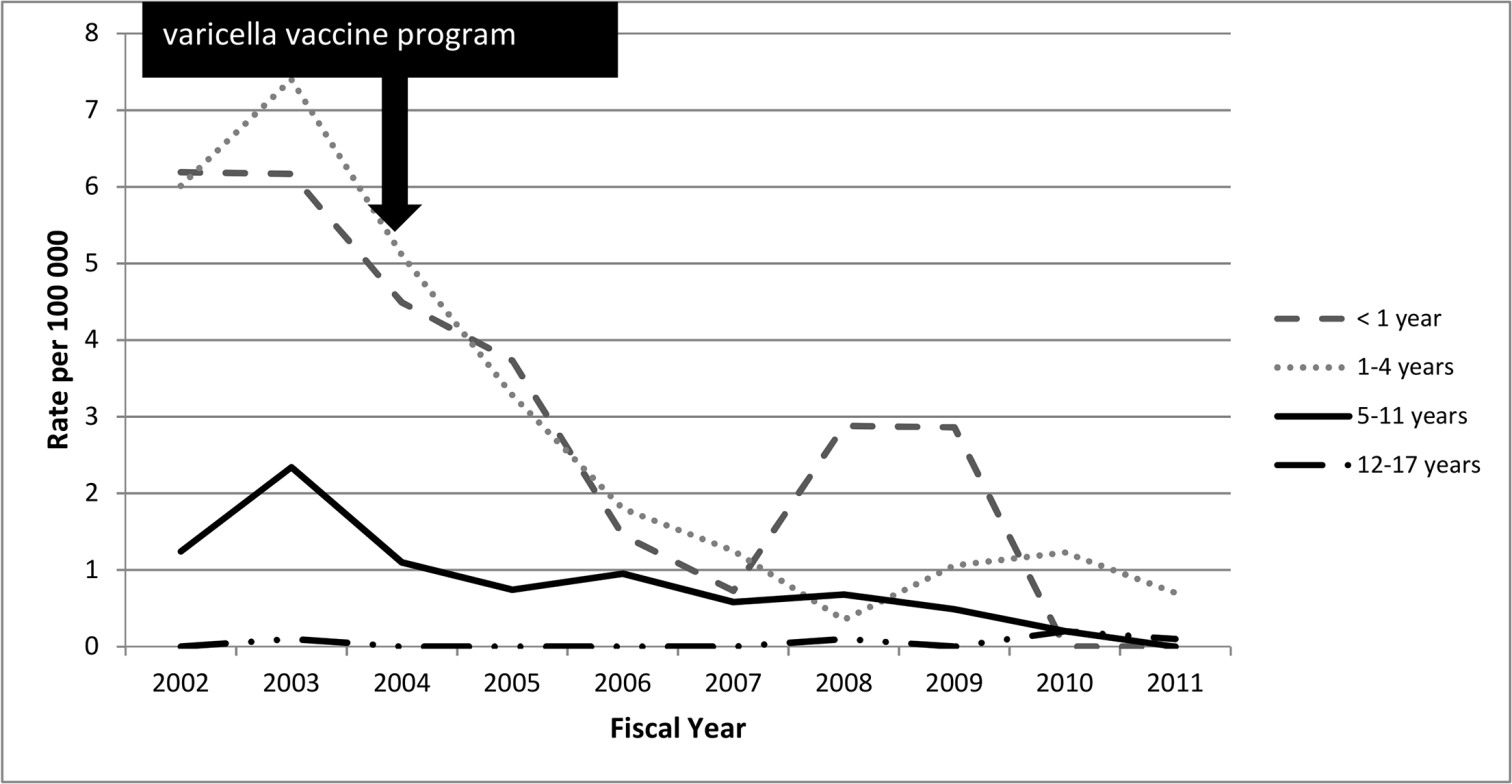
Age-specific varicella physician office visits among Ontario children by fiscal year, 1992–2011.

Age-specific annual declines in varicella office visits were 7.7%, 9.1%, 8.4%, and 8.4% per year among children aged <1 year, 1–4 years, 5–11 years, and ≥12 years, respectively (Fig 2). Likewise, using Poisson regression, rates of varicella office visits, ED visits, and hospitalization all declined significantly over time (p<0.001) for those <1 year and ≥12 years.

Incidence rates of varicella office visits, ED visits, and hospitalizations were greatest in the pre-varicella vaccine era (Table 1). When the incidence of medically-attended varicella in the pre-varicella vaccine, the privately-available varicella vaccine, and varicella vaccine program eras were compared using IRRs and their 95% confidence intervals, office visits and ED visits decreased significantly in all but one comparison (non-significant decrease in physician office visits when the program era was compared to the privately available era) with the greatest decreases occurring in the comparisons of the program era to the pre-vaccine era. Although the IRRs calculated for hospitalizations were less than one for each of the comparisons, none reached statistical significance.

**Table 1:** Comparison of incidence of medically-attended varicella and hospitalization for complicated varicella in Ontario children among pre-varicella vaccine, privately-available varicella vaccine and varicella vaccine program periods.

	Medically-attended varicella, rate per 100,000 per year			Hospitalization for varicella complication, rate per 100,000 per year	
Time Period	Physician Office Visits	ED Visits	Hospitalizations	Varicella-associated SSTI	ICU admission
Pre-varicella vaccine (FY 1992–1998)	1756.77	158.8	7.6	n/a	0.42
Privately-available varicella vaccine(FY 1999–2003)^1^	1174.2	114.1	6.3	2.36	0.47
Varicella vaccine program(FY 2004–2011)	503.6	46.0	2.7	0.72	0.20
Incidence Rate Ratio (95% Confidence Interval) for comparison of privately-available era to pre-varicella vaccine era	0.67(0.62, 0.72)	0.72 (0.57, 0.91)	0.96 (0.32, 2.86)	n/a	1.12(0.02, 71.83)
Incidence Rate Ratio (95% Confidence Interval) for comparison of varicella vaccine program era to privately-available varicella vaccine era	0.43 (0.15, 1.22)	0.40 (0.29, 0.57)	0.43 (0.10, 1.77)	0.31(0.02, 4.27)	0.43(0, 79.70)
Incidence Rate Ratio (95% Confidence Interval) for comparison of varicella vaccine program era to pre-varicella vaccine era	0.29 (0.26, 0.32)	0.29 (0.21, 0.40)	0.41 (0.10, 1.69)	n/a	0.48(0, 97.80)

Notes: 1. For varicella-associated SSTI, privately-available varicella vaccine period consists of FY 2002–2003.

#### b) Trends in complicated varicella infection

There were 290 hospitalizations for varicella-associated SSTIs between FY2002 and FY2011 and 187 varicella ICU admissions between FY1992 and FY2011. Males accounted for a 56.0% of varicella-associated SSTI hospitalizations and 54.6% of ICU admissions. Age-specific incidence of the two complicated varicella outcomes was highest for infants aged <1 year and lowest for adolescents aged 12–17 years. Among infants, the average annualized incidences of varicella-associated SSTI hospitalizations and ICU admissions were 2.77 per 100,000 (FY2002-2011) and 1.01 per 100,000 (FY1992-2011), respectively. In contrast, the corresponding rates were 0.05 and 0.09 per 100,000 for adolescents aged 12–17 years, respectively.

**Fig 3:**
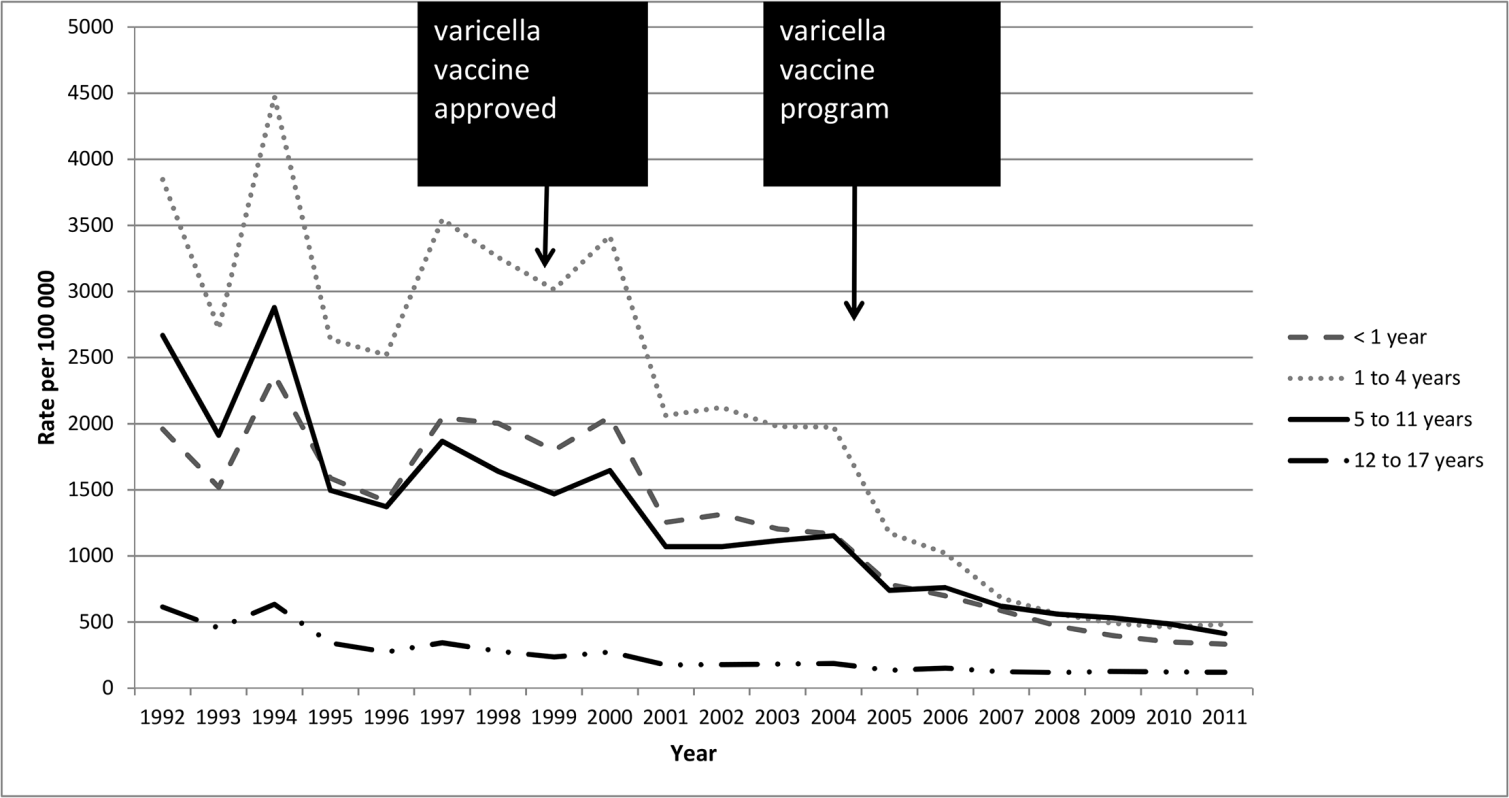
Age-specific varicella-associated SSTI hospitalizations among Ontario children by fiscal year, 2002–2011.

Varicella-associated SSTIs decreased significantly (p<0.001) among all age groups except the 12–17 years group (p = 0.10), a group with few of these admissions during the 10-year observation period, as shown in Fig 3. The highest age-specific incidence of SSTI hospitalization during the study period was 7.40/100 000 (n = 41 cases) in 2003 for children aged 1–4 years. Since 2007, there have been seven or fewer children aged 1–4 years hospitalized with SSTI each year. Likewise, the incidence rate of varicella-associated SSTI admission among infants <1 year dropped from 6.19/100,000 (n = 8) in 2002 to zero in 2010 and 2011 (Fig 3).

Varicella-associated ICU admissions occurred infrequently. However, they decreased significantly over time among those aged <1 year (p < 0.05) but did not significantly change in any other age group (all p>0.05). The incidence rate of varicella-associated ICU admissions among children aged <1 year was greatest in 1998 (2.99/100,000; [n<6]) and lowest in 2002, 2005, 2006 (all zero). For 1–4 year olds, incidence rate of varicella-associated ICU admissions ranged from 1.54/100,000 (n = 9) in FY1999 to 0.17/100,000 in FY2011 (n<6). The overall incidence of complicated varicella admissions declined during the study period. The annual rate of SSTIs and ICU admissions decreased significantly over the ten year period (both p<0.001). Rates during each era are shown in Table 1.

#### c) Shift to older age at infection

Median ages of varicella cases over the entire study period were 5.2, 4.9, and 4.2 years for office visits, ED visits, and hospitalizations, respectively. The median ages increased significantly over time (all p<0.001) in all three settings whereas increases in median age were non-significant for both types of varicella complication. The significant increase in the median age of children with varicella office visits from the pre-vaccine era to the post vaccine era was approximately 11 months. As seen in Table 2, throughout the eras, median ages were older among those seen for ambulatory care (office visits and ED visits) versus among inpatients (hospitalizations, SSTI and ICU admissions).

**Table 2:** Median (IQR) age of varicella cases by clinical setting in the pre-varicella vaccine period, the privately-available varicella vaccine period and varicella vaccine program period.

	Varicella physician office visits	Varicella ED visits	Varicella hospitalizations	Varicella-associated SSTI^1^	ICU^2^ admission
Time Period	Median age (IQR), years				
Pre-varicella vaccine (FY 1992–1998)	5.1 (4.5)	5.0 (5.1)	4.0 (5.3)	n/a	3.8 (5.0)
Privately-available varicella vaccine^3^ (FY 1999–2003)	5.0 (4.3)	4.6 (4.9)	3.5 (4.6)	3.5 (4.2)	3.7 (4.6)
Varicella vaccine program (FY 2004–2011)	6.0 (5.4)	5.2 (5.8)	4.4 (6.3)	4.0 (4.1)	4.3 (7.1)

Notes: 1. SSTI (Skin and soft tissue infection). 2. ICU (Intensive care unit). 3. For varicella-associated SSTI, privately-available varicella vaccine period consists of FY 2002–2003.

### Medically-attended herpes zoster

There were 53,256 incident office visits, 5,904 incident emergency department visits, and 563 incident hospitalizations for herpes zoster among Ontario children aged 5–17 years during the study period. The proportion male was 45.9% for physician office visits, 44.9% for ED visits, and 54.9% for hospitalizations.

Greater average annualized age-specific rates of herpes zoster office visits and ED visits were observed among those aged 12–17 years (137.43/100,000 and 16.19/100,000, respectively) compared to aged 5–11 years (office visits: 128.09/100,000; ED visits: 14.65/100,000). In contrast, the rate of hospitalization was similar between the two age groups (1.72/100,000 for adolescents 12–17 years and 1.78/100,000 for children 5–11 years versus).

**Fig 4:**
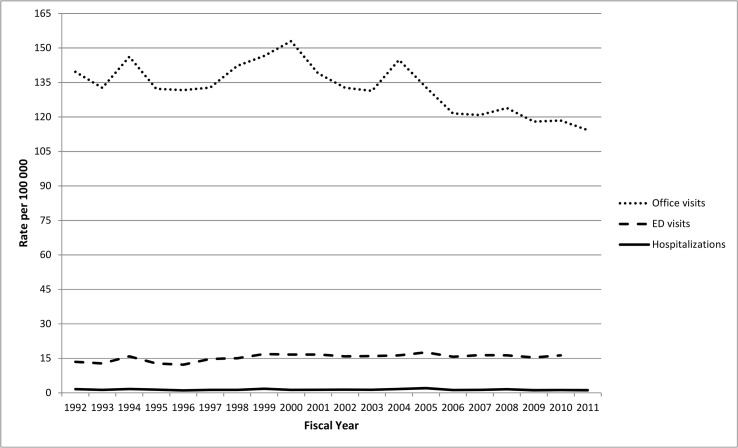
Incidence of herpes zoster physician office visits, ED visits and hospitalizations among Ontario children 5–17 years by fiscal years 1992–2011.

Yearly changes in rates of herpes zoster among children aged 5–17 years varied by clinical setting (Fig 4). During the study period, office visits decreased significantly (p<0.001) and ED visits increased (p<0.001) but the magnitude of the change in ED visits was small (from 12.26 per 100,000 in 1996 to 16.29 per 100,000 in 2008). There was a non-significant decrease in hospitalizations, which occurred very infrequently (p = 0.07).

When the three eras (pre-vaccine, privately-available, and vaccine program) were examined for children aged 5–17 years in all three clinical settings, the IRRs comparing the incidence of herpes zoster in the period of private availability to the pre-vaccine era and the IRRs comparing the the varicella program era to the pre-vaccine period yielded no significant differences.

The secondary analysis of yearly changes in incidence including all children <18 years demonstrated significant decreases in office visits and hospitalizations (p<0.001) but no significant change in ED visits (p>0.05). Comparisons of IRRs across the three periods in all three clinical settings were no different for 0–17 year-olds versus 5–17 year-olds (i.e., no significant changes).

## Discussion and Conclusion

In this large population-based cohort, over 20 years, we demonstrated a significant reduction in pediatric varicella, temporally-associated with Ontario’s publicly-funded varicella immunization program. Further, it appears changes in varicella epidemiology attributable to an immunization program have occurred. These include reduction in complicated disease, a shift to older age at infection (i.e. greater median age), and decreased rates among both program eligible and ineligible age groups (herd effects). The impact of Ontario’s varicella immunization program on pediatric herpes zoster is not clear; although there were no changes in incidence comparing the program periods, ED visits increased significantly over time whereas office visits and hospitalizations decreased, the former significantly.

Although children <1 year and ≥12 years of age were ineligible for routine or catch-up publicly-funded programs during the entire observation period, the annual percent reductions in varicella office visits were similar in these age groups to those in observed in the program eligible age groups (1–4 years and 5–11 years). While we don’t have individual immunization information (see limitations below), disease reductions among program ineligible cohorts, especially those < 1 year of age who are excluded from varicella immunization, imply herd effects.

These findings are consistent with those of an analysis of Ontario reportable diseases data [21] and other major studies of varicella vaccine programs from the United States [1,14, 22, 23], Australia [2], and elsewhere in Canada [3,4], including a preliminary study conducted with Ontario’s health care administrative data two years after the introduction of the publicly-funded program [7]. Rates of medically-attended paediatric varicella have declined since the previous Ontario analysis, whose last year of data was FY2006. For example, Kwong and co-authors reported that 2005–2006 incidence rates of varicella office visits, ED visits and hospitalizations by children aged <1 year were 870/100,000, 147.4/100,000 and 18.6/100,000, respectively. During our last year of study, FY2011 office visits, ED visits and hospitalization visit rates were 332/100,000, 72/100,000 and 8.3/100,000. These reductions suggest ongoing immunization program success. In addition, although office visits decreased non-significantly (based on IRR) between the program and private periods and no IRRs for hospitalizations were significant (non-significant findings may be due to vaccine use and early impact on severe [i.e. hospitalized] cases of varicella during the private period), we found the reductions in incidence of medically-attended disease were of greater magnitude in publicly funded varicella vaccine program era versus the privately-available varicella vaccine era, where less population impact was detected. Cost and public perception of varicella may have contributed to limited vaccine uptake (see vaccine sales data below), and subsequent impact, during the privately-available period.

Regarding complications, prior to the implementation of varicella immunization programs in all Canadian provinces, 58% of GAS necrotizing fasciitis cases were associated with varicella infection based on active surveillance of Canadian pediatricians from 2001 to 2003 [24]. The ability to prevent necrotizing fasciitis was an impetus for routine childhood varicella vaccination programs and we have shown a decline in varicella-associated SSTIs with the introduction of publicly-funded varicella vaccine in Ontario.

The relationship between varicella vaccine and subsequent herpes zoster among recipients, as well as herpes zoster in the population at-large, is not well understood. While some have observed an increase in herpes zoster among immunized or immunization-eligible children [14], most have not [1, 11,–13]. Our results provide reassurance that there has not been an increase in the incidence of pediatric herpes zoster associated with the introduction of publicly-funded varicella vaccine. The increase in ED visits among 5–17 year-olds, which was not seen in the secondary analysis that included children under 5 years of age warrants ongoing study of herpes zoster, a condition that likely requires a longer observation period to determine if its incidence has been altered. Further, there may be a role for chart-review validation of pediatric herpes zoster cases found in administrative databases to determine if the cases are clinically compatible with herpes zoster.

There are limitations to the present study. Medically-attended disease does not represent the true extent of pediatric varicella [25] and we were unable to accurately determine incidence of varicella. At the time of study, we could not use health care administrative data to assess the receipt of varicella immunization and Ontario does not have a registry of individual-level immunization information. As such, we could neither assess immunization coverage nor effectiveness.

Our conclusions with respect to herd immunity rely on the assumption that varicella vaccine is given to the target age group through the publicly-funded program. This assumption is supported by estimates of coverage and vaccine sales data. First, immunization coverage of school pupils is assessed annually through aggregate reports from local public health units. In the 2012–13 school year, 77.8% of 5-year-olds (born in 2006 and 2007, i.e., program eligible) were estimated to have a received a single dose of varicella vaccine [26]. This may be an underestimate as prior to 2014 varicella was not among the antigens for which documentation of immunization status is legally required for school attendance [27, 28]. Second, we obtained varicella vaccine distribution and sales data from the Ontario Government Pharmacy and one manufacturer for the last full year in which Ontario had a single dose varicella program (2010). In 2010, 156,044 publicly-funded varicella doses were distributed [29] and the population of children aged 1 year (born 2009, many of whom would reach 15 months of life in 2010) was similar (n = 142,128) [16]. Merck, one of two manufacturers that supply varicella-containing vaccine in Ontario, provided us with data demonstrating that privately-purchased varicella vaccine now accounts for a very small proportion of their varicella vaccine sales. While in 2004, 46.5% of 128,013 varicella doses sold by Merck were on the private market, that proportion dropped to 1% of 128,139 doses in 2010 (personal communication, Catherine Paquette, Manager, Public Health Policy and Government Relations, Vaccines, Merck, May 2014). Viewed together, coverage estimates, vaccine distribution, and vaccine sales data imply receipt of vaccine by the targeted age group through the publicly-funded program. This supports our conclusions regarding direct and herd effects from the program.

While the methodology for determining ICU admissions has been validated [18], the diagnostic combinations used to study hospital admission for varicella-associated SSTIs (varicella plus *one* of cellulitis, necrotizing fasciitis, or non-pharyngitis group A streptococcal infection) were not. Further, any study that relies on healthcare administrative data is subject to misclassification due to coding, or other, errors. Finally, we have been careful to state that we have observed an association between Ontario’s varicella immunization program and reductions in medically-attended varicella, not necessarily a causal relationship. As others have described, inferences drawn from statistical comparisons of rates between pre-policy and post-policy periods, such as ours, assume negligible impact of temporal trends [30]. We cannot rule out other factors that may have contributed to changing varicella epidemiology.

The large sample size and long observation period are important strengths of our study. We used a much longer vaccine program era than previous assessment of varicella vaccine impact in Ontario and were able to demonstrate further reductions in medically-attended varicella [7]. Healthcare administrative data allowed us to study complications of varicella as well as herpes zoster, both of which are not possible when public health reports of varicella are analyzed, due to incomplete clinical information for varicella cases and because herpes zoster is not a legally reportable disease [21].

Reductions in pediatric varicella suggest immunization program success in Ontario. However, future analyses will be needed to assess the impact of the two-dose program, especially on varicella outbreaks, and to obtain better understanding of the relationship between childhood varicella immunization and herpes zoster.

## Supporting Information

S1 Table ICD-10 diagnostic code for hospitalization with varicella-associated SSTI and OHIP codes for ICU admissions. (DOCX)
