# Correction: Twenty Years of Medically-Attended Pediatric Varicella and Herpes Zoster in Ontario, Canada: A Population-Based Study

**DOI:** 10.1371/journal.pone.0138397

**Published:** 2015-09-14

**Authors:** The PLOS ONE Staff

There are errors in the Funding section. The correct funding information is as follows: This study was supported by the Institute for Clinical Evaluative Sciences (ICES) and Public Health Ontario (PHO), which are funded by annual grants from the Ontario Ministry of Health and Long-Term Care (MOHLTC). The opinions, results and conclusions reported in this paper are those of the authors and are independent from the funding sources. No endorsement by ICES, PHO, or the Ontario MOHLTC is intended or should be inferred.

The images for figures Figs 2 and 3 are swapped. The image for Fig 2 should be Fig 3 and the image for Fig 3 should be Fig 2. The figure captions are correct. The publisher apologizes for the error. Please see the corrected Figs 2 and 3 here.

Fig 1 is incorrect. Please see the corrected Fig 1 here.

**Fig 1:**
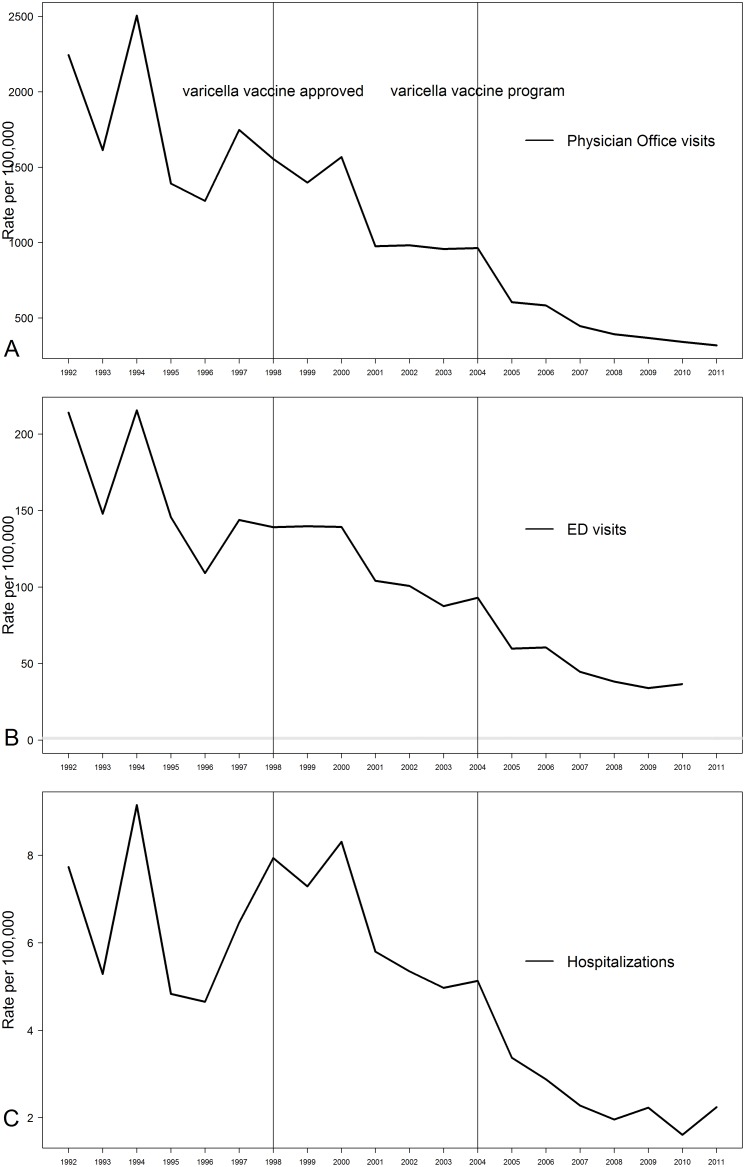
Incidence of varicella a) physician office visits (n = 600,208), b) ED visits (n = 55,472), and c) hospitalizations (n = 2,701) among Ontario children by fiscal years 1992–2011.

**Fig 2:**
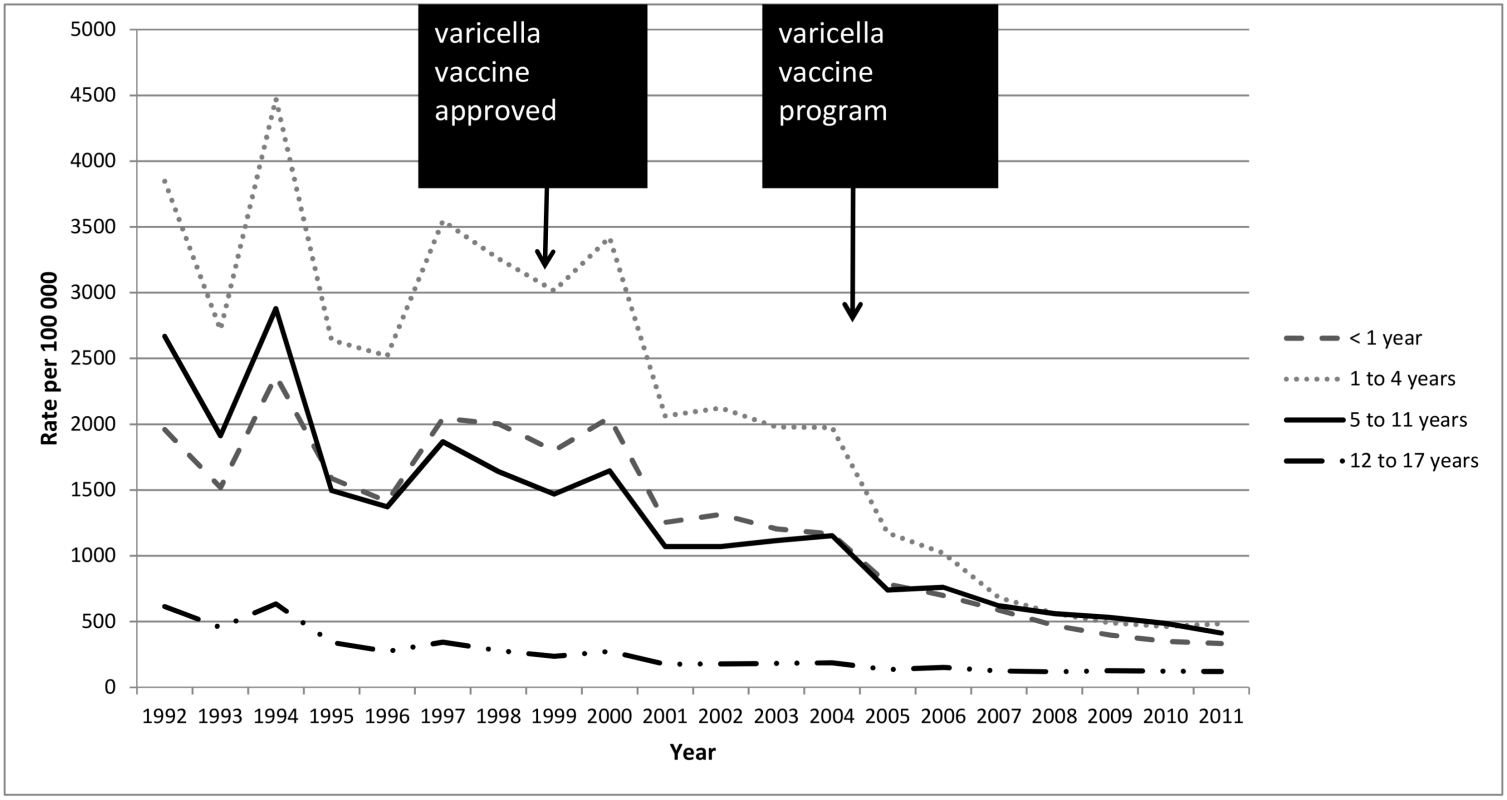
Age-specific varicella physician office visits among Ontario children by fiscal year, 1992–2011.

**Fig 3:**
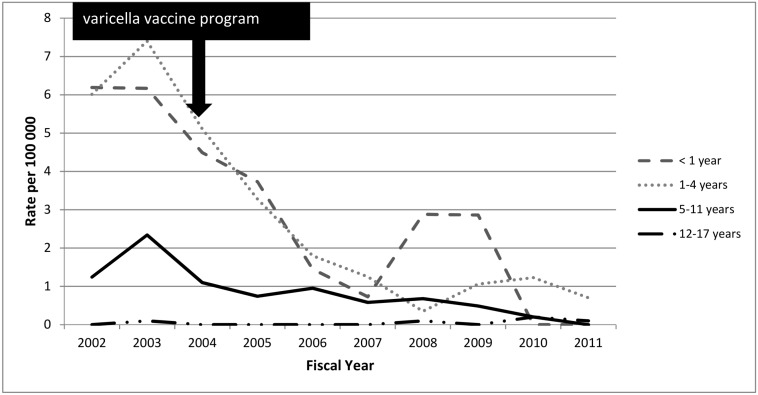
Age-specific varicella-associated SSTI hospitalizations among Ontario children by fiscal year, 2002–2011.

There was information omitted from the Acknowledgements. The complete Acknowledgements is as follows: These datasets were linked using unique encoded identifiers and analyzed at ICES. Parts of this material are based on data and information compiled and provided by the Canadian Institute for Health Information (CIHI). However, the analyses, conclusions, opinions and statements expressed herein are those of the author, and not necessarily those of CIHI. The authors wish to acknowledge the assistance of Danijela Draganic, Steven Janovsky and Lennon Li, Public Health Ontario, for assistance with preparation of figures for publication.
