# Supplementary material for: Twenty Years of Medically-Attended Pediatric Varicella and Herpes Zoster in Ontario, Canada: A Population-Based Study
Source: PLoS One. 2015 Jul 15;10(7):e0129483. doi: 10.1371/journal.pone.0129483 (PMC4503773; doi:10.1371/journal.pone.0129483)
Supplement: S1 Table — (DOCX) [file pone.0129483.s001.docx]

**S1 Table. ICD-10 diagnostic code for hospitalization with varicella-associated SSTI and OHIP codes for ICU admissions.**

| **Secondary Soft Tissue Infection Associated with varicella** | | |  |  |
| --- | --- | --- | --- | --- |
| Cellulitis with varicella |  | L03 and coexistent  B01.0-B01.9 |  |  |
| Necrotizing fasciitis with varicella |  | M726 and coexistent  B01.0-B01.9 |  |  |
| Group A streptococcal infection with varicella |  | B01.0-B01.9 but exclude  coexistent B02.0-B02.9 (herpes zoster**) and** coexistent  B95.0 (Group A streptococcus), or  B95.4 (other streptococcus),  or B95.5 (streptococcus non-specificed) or A40.0 (sepsis due to streptococcus Group A) or A40.8 (other streptococcal sepsis), A40.9 (streptococcal sepsis unspecified) but exclude coexistent J02.0 (streptococcal pharyngitis) |  |  |

Intensive care unit admissions:

Varicella ICD 10 code (B01.0, B01.1, B01.2, B01.8, B01.9) **and** OHIP billing code for ICU care (e.g. mechanical ventilation). OHIP billing codes: G557, G558, G559, G400, G401, G402, G405, G406, G407,C101
